# Analgesic Effects, Birth Process, and Prognosis of Pregnant Women in Normal Labor by Epidural Analgesia Using Sufentanil in Combination with Ropivacaine: A Retrospective Cohort Study

**DOI:** 10.1155/2022/1228006

**Published:** 2022-08-29

**Authors:** Lijing Mao, Xiaoxiao Zhang, Jing Zhu

**Affiliations:** ^1^Department of Obstetrics, Nantong Maternal and Child Health Hospital, Nantong 226000, China; ^2^Anesthesiology Department, Nantong Maternal and Child Health Hospital, Nantong 226000, China

## Abstract

**Objective:**

The objective is to evaluate the analgesic, labor, and prognostic effects of patient-controlled epidural analgesia (PCEA) versus sufentanil in conjunction with ropivacaine in normal labor.

**Methods:**

Sixty pregnant women who had a normal delivery at our hospital between February 2019 and April 2021 were included. Pregnant women were arbitrarily assigned to a control group and a research group. Pregnant women in the control group received lidocaine analgesia and PCEA with sufentanil combined with ropivacaine in the research group. Satisfaction with care, fetal umbilical artery blood flow, VAS score, labor and bleeding, neonatal Apgar score and incidence of adverse events were analyzed.

**Results:**

First, we made a comparison of satisfactory performance of nursing care. The satisfaction rate of the research group was 100.00%, compared to 83.33% for the control group. Nursing satisfaction was higher in the research group, and the difference was statistically significant (*P* < 0.05). Following analgesia, PI, RI, and S/D values of umbilical artery blood flow were lower in the research group than those in the control group, but the difference was not statistically significant (*P* > 0.05). The VAS scores at 10 min, 20 min, and 30 min were found to be lower in the research group than in the control group after analgesia, and the difference was statistically significant (*P* < 0.05). Bleeding was significantly lower in the research group for all stages of labor, and the difference was statistically significant (*P* < 0.05). Apgar scores at 1 minute, 5 minutes, and 10 minutes postpartum were greater in the research group than in the control group, and the difference was statistically significant (*P* < 0.05). As a final note, the incidence of pruritus, hypotension, respiratory depression, nausea, and vomiting was found to be lower in the research group than in the control group, and the difference was statistically significant (*P* < 0.05).

**Conclusion:**

PCEA with sufentanil coupled with ropivacaine was used to perform labor analgesia. With significant reduction in maternal pain and assurance of labor, ropivacaine combined with sufentanil epidural labor analgesia did not reduce fetal umbilical artery blood flow without extended labor. It could not affect the labor process or the safety of the fetus, which is safe for the mother and fetus.

## 1. Introduction

In the process of delivery, pregnant women have severe pain due to uterine contraction and uterine dilatation, and many pregnant women choose cesarean section for fear of labor pain, which has an adverse impact on the physical and mental health of mothers and infants [1]. Pregnant women are often accompanied by bad emotions such as tension and fear in the process of delivery, and labor pain will aggravate maternal tension and fear, which promote each other and form a vicious circle, and the final result is the lack of physical strength. A large amount of maternal consumption results in prolonged labor. Fetal distress may even occur, affecting maternal and infant safety. With the improvement of people's living standards, people put forward higher requirements for the comfort of medical activities. To this end, we strive to provide patients with “patient-centered” comfortable medical services by strengthening service measures and optimizing the service environment [2]. With the development of anesthetic technology and the improvement of maternal requirements for labor analgesia, epidural labor analgesia is widely used in parturition, thus effectively relieving severe pain in the process of delivery [1, 2]. Epidural labor analgesia during the latent period did not prolong the duration of labor nor did it increase the rate of cesarean section. According to the 2016 version of the consensus of labor analgesia experts, labor analgesia begins with the need for pain relief, not the size of uterine dilatation [3]. For fear of prolonging the second stage of labor, some hospitals stop using analgesic pumps when the uterine mouth is full, but the latest research indicates that the whole process of labor analgesia does not change the delivery outcome, on the contrary, it can enhance the maternal satisfaction [4]. Labor analgesia can effectively reduce the fear of parturient, get sufficient rest in the whole process of labor, avoid unnecessary physical and oxygen consumption, and help to enhance the oxygenation function of the fetus, thus enabling the parturient to establish self-confidence. It has a positive effect on the physical and mental health of mothers and infants [5].

Normal physiological delivery is assigned into three stages of labor. The nature of labor pain in the first and second stages of labor is different, and the reason lies in the mechanism, source, and innervation of pain [6]. Ropivacaine hydrochloride injection is a long-acting amide local anesthetic with low cardiotoxicity and neurotoxicity. Low concentration ropivacaine has the advantage of separating sensorimotor block and rarely passes through the placenta [7]. When adopted in epidural space, the motor block effect of ropivacaine was weaker compared to other amide local anesthetics [8]. Sufentanil is a *μ*-opioid receptor agonist with good analgesic effect, which can cooperate with amide local anesthetics in epidural anesthesia to further reduce the dosage of local anesthetics, thus reducing the block of local anesthetics on the movement of lower limbs. The analgesic effect is faster and lasts longer, so as to achieve walking labor analgesia [9, 10]. Ropivacaine is a new type of long-acting amide local anesthetic, which blocks the conduction of excitement by inhibiting Na+ channel. 0.1% ropivacaine had no blocking effect on the movement of human lower limbs. Ropivacaine is a left-handed stereoisomer. When local anesthetic poisoning occurs, the success rate of cardiopulmonary resuscitation is much higher compared to bupivacaine. In epidural anesthesia, the concentration of local anesthetic in cerebrospinal fluid is relatively low due to the blocking effect of arachnoid. In animal experiments, ropivacaine can cause a transient, reversible ascension to the medullary transmembrane channel [11]. Bupivacaine can enhance the permeability of fat-soluble membranes, and the effect of ropivacaine is relatively mild, so the toxicity of bupivacaine is stronger [12]. Animal model experiments show that the distribution of local anesthesia was different between intrathecal and epidural anesthesia, and the bioavailability of intrathecal ropivacaine decreased after epidural injection [13]. There exhibits no significant difference in sensory block between opium and Bubby. The motor block time and analgesic effect of the two drugs are similar. Studies have indicated that the analgesic effect of Roopa is weaker compared to Bubby. In continuous epidural anesthesia, the recovery of sensory block was slower in patients using ropivacaine than in patients using bupivacaine [14]. Many studies have indicated that the longer the duration of analgesia, the higher the incidence of motor block with bupivacaine and ropivacaine [15]. Ropivacaine combined with opioids, such as fentanyl or sufentanil, can not only enhance the analgesic effect and prolong the analgesic time but also does not increase the incidence of motor block. Therefore, in the process of delivery, ropivacaine combined with sufentanil or fentanyl for maternal analgesia can promote the effect of maternal analgesia and strengthen maternal satisfaction rate [16]. This study focuses on the effects of PCEA combined with sufentanil and ropivacaine on maternal analgesia, labor process, and delivery outcome, in order to provide a theoretical basis for the clinical application of labor analgesia.

## 2. Patients and Methods

### 2.1. Normal Information

Sixty patients with natural delivery in our hospital from February 2019 to April 2021 were enrolled. The patients were randomly divided into control group and research group. The former received sufentanil for analgesia, whereas the latter received sufentanil combined with ropivacaine for PCEA. In the control group, the patients were aged from 20 to 34 years old, with an average of 27.23 ± 1.63 years old; In the research group, the age ranged from 20 to 35 years, with an average of 27.42 ± 1.58 years. There exhibited no statistical significance in the general data. This study was permitted by the Medical Ethics Association of our hospital, and all patients noticed informed consent.

Selection criteria: (1) According to the American College of Anesthesiologists (ASA) grade I∼II. (2) All belong to full-term singleton parturient, head position. (3) The age was 20–35 years old and the weight was 60–85 kg. (4) There were no contraindications of intraspinal anesthesia and obstetrics. (5) Parturients voluntarily accept intrathecal labor analgesia.

Exclusion criteria: (1) Patients with abnormal coagulation function and easy bleeding. (2) Systemic infection increased intracranial pressure. (3) There are gestational diabetes, placenta previa, preeclampsia or eclampsia, heart disease, fetal intrauterine growth abnormalities, and other complications. (4) Have a history of mental illness, epilepsy, hysteria, and cannot cooperate normally. (5) Those who were evaluated by the obstetrician could not give birth through the vagina. (6) The parturients and their families refused to accept the pain relief of intraspinal delivery.

### 2.2. Treatment Methods

When regular uterine contractions appear, the following treatments were implemented: vital signs routine monitoring, upper extremity venous access opening, compound sodium chloride solution infusion, fetal heart rate monitoring, and uterine contraction intensity detection. When the cervix of the control group was enlarged to 3 cm, epidural puncture was performed in the L2-3 space and a 3.5 cm tube was placed on the cephalic side, no blood or cerebrospinal fluid was withdrawn, and the test dose of 1.0% lidocaine was injected 3 ml, observe for 5 min to exclude intravascular catheter and subarachnoid administration. The research group was given sufentanil combined with ropivacaine for PCEA, and the loading dose of 10 ml (0.125% ropivacaine + 0.4 *μ*g/ml sufentanil) was injected uniformly through the epidural catheter. If the VAS score (visual analog score) of the parturient was still greater than 3 after the first dose of 15 min, the case was withdrawn from the study. After epidural analgesia took effect, PCEA pump (100 ml) was connected, PCEA regimen: background dose 10 ml/h, self-controlled dose 8 ml, locking time 15 min. Instruct the parturient when VAS ≥ 4, press the drug by themselves, and stop the drug administration after the uterine mouth is fully opened.

### 2.3. Observation Indicator

#### 2.3.1. Satisfaction

After consulting the literature and expert discussion, we designed patients' follow-up satisfaction, a total of 10 items, and recorded patients' satisfaction with follow-up management mode, health education, medical and nursing service, appointment registration process [17]. It is assigned into four dimensions: very satisfied, satisfied, general, and dissatisfied. Satisfaction rate = very satisfaction rate + satisfaction rate + general rate.

#### 2.3.2. Fetal Umbilical Artery Blood Flow

The superior SONIXTABLET ultrasound instrument was used for examination. The probe frequency was set to 2.0∼5.0 MHz, the sampling volume was 2 mm, and the angle between the pulsed Doppler sampling line and the blood vessel was less than 20°. The image of the umbilical artery was captured near the umbilical foramen on the side of the fetus. After finding the corresponding artery, when three consecutive regular waveforms appear on the ultrasound screen, the S and D values can be measured by freezing the image, and the resistance index RI [RI = (*S* − *D*)/*S*], Pulsatility index Pi [PI = 2 (*S* − *D*)/(*S* + *D*)] and *S*/*D* value can be calculated automatically. All monitoring operations and data recording were performed by the same attending ultrasound doctor with rich clinical experience.

#### 2.3.3. VAS Scoring

VAS for short draws a horizontal line from 0 to 10, with 0 for painless, and 10 for severe pain; The numbers in the middle represent different degrees of pain. Pregnant women write numbers and score according to their subjective pain feelings. 0–3 points indicate mild pain, 4–6 points indicate moderate pain, and 7–10 points indicate severe pain.

#### 2.3.4. Parturient Stage of Labor

The following data were collected: the time of the first stage of labor, the time of the second stage of labor, and the amount of blood loss.

#### 2.3.5. Apgar Scoring

The neonatal Apgar score was observed and recorded. According to the Apgar score, according to the five signs of skin color, heart rate, respiration, muscle tone, and reflex after birth, normal newborns were normal with 2 points in each item, no respiratory depression, mild asphyxia under 7 points, and severe asphyxia below 4 points.

#### 2.3.6. Incidence of Adverse Reactions

The adverse reactions such as pruritus, fever, hypotension, respiratory depression, nausea, and vomiting were observed and recorded.

### 2.4. Statistical Analysis

SPSS23.0 statistical software was adopted to process the data. The measurement data were presented as ($\bar{x}$ ± *s*). The group design *t*-test was adopted for the comparison and the analysis of variance was adopted for the comparison between multiple groups. Dunnett's *t*-test was adopted for comparison with the control group. The counting data were presented in the number of cases and the percentage, *χ*^2^ test was adopted for comparison between groups, and bilateral test was employed for all statistical tests.

## 3. Results

### 3.1. Comparison of Nursing Satisfaction

**Figure 1:**
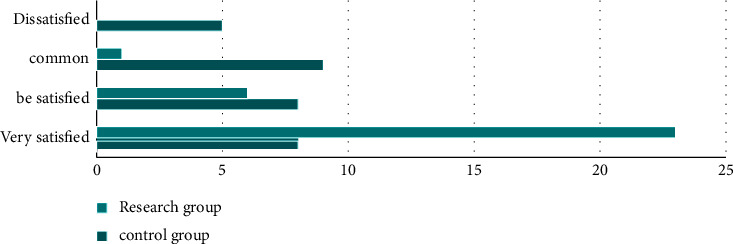
Comparison of the incidence of adverse reactions.

First of all, we compared the nursing satisfaction. The research group was very satisfied in 23 cases, satisfactory in 6 cases and general in 1 case, with a satisfaction rate of 100.00%; In the control group, 8 cases were very satisfied, 8 cases were satisfied, 9 cases were general, and 5 cases were dissatisfied, the satisfaction rate was 83.33%; The nursing satisfaction of the research group was higher compared to the control group, and the difference was statistically significant (*P* < 0.05). All the data results are indicated in Figure 1.

### 3.2. Comparison of Fetal Umbilical Artery Blood Flow

Secondly, we compared the fetal umbilical artery blood flow. Before analgesia, there exhibited no significant difference (*P* > 0.05); After analgesia, the PI, RI, and *S*/*D* values of umbilical artery blood flow in the research group were lower than those in the control group, but the difference was not statistically significant (*P* > 0.05); All the data results are indicated in Table 1.

### 3.3. VAS Score Comparison

Secondly, we compared the VAS scores. Before analgesia, there exhibited no significant difference (*P* > 0.05); after analgesia, the VAS scores decreased. The VAS scores at 10 min, 20 min, and 30 min were lower compared to the control group, and the difference was statistically significant (*P* < 0.05). All data results are indicated in Table 2.

### 3.4. Comparison of the Stage of Labor and the Amount of Bleeding

Secondly, we compared the stage of labor and the amount of bleeding. The first stage of labor, the second stage of labor, the third stage of labor, and the amount of bleeding in the research group were lower compared to the control group, and the difference was statistically significant (*P* < 0.05). All the data results are indicated in Table 3.

### 3.5. Comparison of Neonatal Apgar Score

Secondly, we compared the Apgar scores of newborns. The 1 min, 5 min, and 10 min Apgar scores of newborns in the research group were higher compared to the control group, and the difference was statistically significant (*P* < 0.05). All the data results are indicated in Table 4.

### 3.6. Comparison of the Incidence of Adverse Reactions

**Figure 2:**
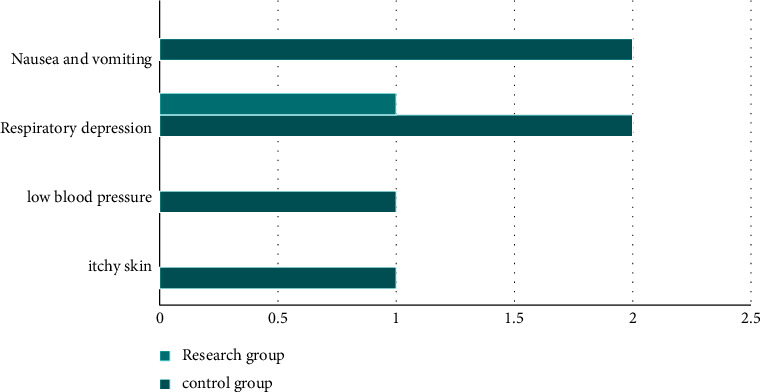
Comparison of the incidence of adverse reactions.

Finally, we compared the incidence of adverse reactions. The incidences of skin pruritus, hypotension, respiratory depression, nausea, and vomiting in the research group were lower compared to the control group, and the difference was statistically significant (*P* < 0.05). All the data results are indicated in Figure 2.

## 4. Discussion

Pain in labor is a common physiological phenomenon, which accompanies the whole process of delivery [16]. According to the traditional view, when the uterine orifice is enlarged to 3 cm, the analgesic effect of labor in the spinal canal is the best. Early application of labor analgesia will reduce the intensity of uterine contraction, prolong the interval of uterine contraction, slow down the speed of uterine dilatation, and prolong the process of labor. However, in the process of clinical practice, it is found that many pregnant women have severe labor pain before entering the active stage. During the latent period of labor, fentanyl was injected into the epidural space of parturients, and the study indicated that there exhibited no difference in labor time and delivery outcome between labor analgesia and active labor analgesia [17]. Under the premise of ensuring medical safety, we pursue medical comfort and humanization. Make patients feel psychological and physical pleasure, no pain and no fear in the whole process of seeking medical treatment. As an important means of comfortable diagnosis and treatment in obstetrics, painless delivery is becoming more important. In China, in order to reduce labor pain, the main measure is to implement epidural painless delivery. According to authoritative data released by the World Health Organization (WHO) in 2010, the cesarean section rate in China is 46.2% (ranking first in the world), which is much higher than the upper limit of WHO cesarean section rate: 15% (of which 11.7% without surgical indication) [18]. The high rate of cesarean section has become not only a medical problem faced by the medical profession but also a serious “public health problem” faced by our country. Among the social factors of cesarean section, maternal inability to bear labor pain is the first cause [19]. In addition, some studies have indicated that labor analgesia can reduce the rate of cesarean section, so the promotion of labor analgesia has important social significance to change the current situation of high cesarean section rate in China [20]. This study focuses on the effects of PCEA combined with sufentanil and ropivacaine on maternal analgesia, labor process, and delivery outcome, in order to provide a theoretical basis for the clinical application of labor analgesia.

Traditional high concentration local anesthetic epidural labor analgesia can effectively relieve labor pain, but has a certain blocking effect on uterine contraction and lower limb motor function [21]. The first stage of labor: from regular uterine contractions to full opening of the uterine mouth. The parturient women in the first stage of labor generally take 7–13 hours, while the parturients generally take 6–8 hours. The first stage of labor consists of the latent period and the active stage. In the first stage of labor, uterine contraction occurs, uterine muscle fibers elongate or even tear, and the decline of fetal head will lead to the dilation of the lower segment of uterus and cervix. The pain in the first stage of labor comes from uterine contraction and dilatation of the lower segment of the uterus and cervix, while the innervation of the uterine body and cervix comes from the visceral sensory nerves. The latent period of labor pain is usually dominated by T11-12, while the active phase is introduced through T10-11. The nature of labor pain in the first stage of labor is unclear and the location is uncertain, which belongs to the category of “visceral pain.” The intensity of labor pain in the first stage of labor is closely related to the intensity of uterine contraction, uterine pressure, and other factors, and the labor pain reaches the peak when the cervix is opened and 7∼8 cm. The second stage of labor starts from the opening of the uterine orifice to the end of fetal delivery [22]. The pain in the second stage of labor comes from the expansion, pull and tear of the skin, muscle, fascia, and other tissues of the lower birth canal. The nerve conduction of labor pain in the second stage of labor comes from the lower birth canal, and the pain signal is transmitted along the pudendal nerve S234 and quickly uploaded to the nerve center. The nature of the second stage of labor pain is knife-like sharp pain, the pain site is relatively fixed, generally near the vagina, perineum, and rectum, belonging to the category of “body pain.” Due to the strong uterine contraction in the second stage of labor, some scholars believe that this stage is the coexistence of “visceral pain” and “physical pain.” The labor pain in the third stage of labor comes from cervical dilatation and uterine contraction during placental delivery [23]. Therefore, the ideal epidural labor analgesia requires that the block level be controlled at T10∼L1 in the first stage of labor, and at S234 in the second and third stages of labor. In this way, we can achieve the goal of blocking only the sensory nerve without affecting the motor nerve, so as to achieve the desire to eliminate labor pain without affecting uterine contraction. It should be noted that when the block level of epidural labor analgesia reaches T5, the intensity and frequency of uterine contraction decrease remarkably [24].

The ideal labor analgesia should have the following characteristics: convenient administration, quick effect, safe and reliable effect, can ensure the sobriety of the parturient and has little influence on the parturient, is not easy to pass through the placental barrier to affect the fetus, and only produces pain block, does not block the motor nerve, does not affect the maternal force and uterine contraction during delivery, and can be used to assist cesarean section at any time [25]. Medical experts and scholars have also been looking for methods of labor analgesia that meet these requirements. In 1847, Dr. Simpson used chloroform for labor analgesia. In 1920, a series of methods such as low epidural block and sacral block were used for labor analgesia. Epidural block was first used for labor analgesia by American physicians Graffagnin and Sevler in 1938. Epidural labor analgesia is very popular because of its definite analgesic effect, little influence on mother and infant, little influence on the course of labor, sober cooperation of parturient, and satisfying operation when the emergency needs to be converted to cesarean section during delivery. In 1979, Dr. Revil put forward epidural block as the most effective method of labor analgesia in Europe. Continuous epidural administration, patient-controlled analgesia (PCA) technology, and the use of new drugs make its application have a broader space, so that epidural labor analgesia has become the main means of obstetrical labor analgesia. The application of PCEA in labor analgesia was first reported in 1988 [26]. Epidural labor analgesia can not only effectively reduce maternal pain and meet the requirements of medical comfort but also can be combined with other disciplines. Some scholars use epidural labor analgesia combined with psychotherapy, the effect is obvious. The rate of cesarean section is greatly reduced [27].

Ropivacaine is a new type of amide with long-acting local anesthesia. It has a pure *S* (-) mirror structure, and its chemical structure is similar to that of bupivacaine and mepivacaine, except that the side chain of nitrogen hexane is replaced by propyl [28]. However, there is no consensus on whether PCEA administration mode should be combined with continuous background infusion. Scholars have found that continuous infusion of PCEA combined with background will lead to an increase in anesthetic dosage, but there is no further improvement in analgesic effect and maternal satisfaction, and even affect the process of labor [29]. However, more studies hold the opposite view, believing that PCEA combined with background infusion has a definite analgesic effect without increasing the anesthetic dosage [30, 31]. The differences in research methods, drug types, and drug doses are the possible reasons for the differences in the research results. Other scholars believe that the duration of epidural labor analgesia has a circadian rhythm, and the action time of the same dose of drugs in the daytime is longer than that at night [32]. It also suggests that we should consider the effect of circadian rhythm on analgesia time, so as to make the research groups more comparable. The effect of circadian rhythm may be one of the possible factors contributing to the inconsistency of the above results. Recently, some scholars have put forward a new point of view on the background infusion mode. Through the latest computer integrated patient-controlled epidural analgesia (CI-PCEA) technology, the background infusion rate can be adjusted actively according to the individual needs of pregnant women [33]. If the puerperal presses PCEA1 to add drugs for the first hour, the background infusion speed is automatically adjusted from 0 to 5 mL/h; If the puerperal presses 2 times for 3 times, the background infusion speed is automatically adjusted to 10 mL/h or 15 mL/h; If the puerperal presses 0 times in the first hour, the background infusion rate will automatically decrease 5 mL/h. The researchers believe that compared with PCA without background infusion, CI-PCEA not only does not increase the dosage of anesthetics but also enhances maternal satisfaction with analgesia [34]. The authors changed the continuous infusion dose of 12 mL/h (0.0625% bupivacaine + 2 *μ*g/mL fentanyl) into a single epidural injection of 6 mL/30 min, which was named regular intermittent epidural administration (PIEB). Through regular intermittent epidural administration, if there is insufficient analgesia, the parturient can be treated with PCEA compressions, and if it cannot be relieved, a single epidural injection of 0.125% bupivacaine is required by the anesthesiologist to rescue [35]. The study found that compared with continuous background infusion of PCEA, there exhibited no significant difference between PIEB and its analgesic effect, but the number of maternal PCEA pressing was less, the dosage of anesthetics was remarkably reduced, and the satisfaction of maternal analgesia was promoted. In view of the different sources of pain in the first and second stages of labor, that is, labor pain in the latent period of the first stage of labor is usually controlled by T11-12, while in the active stage, it is transmitted through T10-11, and the pain signal of the second stage of labor is transmitted along the pudendal nerve S2hyd4 and quickly uploaded to the nerve center. Some scholars have explored the dual-tube epidural labor analgesia, puncturing the two-point T12-L1 and L4-5 space and inserting the epidural catheter respectively. Analgesics are given at the upper point in the first stage of labor, and drugs are given at the lower point in the second stage of labor. According to the different sources of pain in the process of labor, pain is given a “precise blow” [36]. The results indicate that this method can not only strengthen the satisfaction of parturient but also reduce the rate of lateral episiotomy and improve the long-term quality of life of parturients.

Although the side chain of nitrogen hexane was replaced by propyl, the potency of ropivacaine decreased. At present, PCEA is mostly adopted in intraspinal block labor analgesia with low concentration local anesthetics combined with analgesics. PCEA is more individualized, which can meet the analgesic needs of different parturients and reduce the dose [36, 37]. The ratio of anesthetic potency between ropivacaine and bupivacaine is about 5 : 8, but it also has some characteristics, such as inherent vasoconstrictor effect, lower protein binding rate, higher clearance rate, and shorter elimination half-life. All these characteristics enable ropivacaine to avoid drug accumulation during multiple injections, thereby reducing the risk of systemic poisoning and greatly reducing cardiotoxicity and neurotoxicity [37]. In addition, the significant advantage of ropivacaine at low concentration is the separation of sensory and motor block, which makes it analgesic without affecting uterine contraction and movement, and is more suitable for obstetric anesthesia. Some scholars have confirmed that low concentration ropivacaine for epidural labor analgesia has no advantage compared with bupivacaine in analgesic effect and effect on mother and infant. Some scholars observed 40 parturients who underwent epidural labor analgesia. The two groups received continuous epidural infusion of 0.0625% ropivacaine + 2 *μ*g/mL fentanyl and 0.0625% bupivacaine + 2 *μ*g/mL fentanyl, respectively. The results indicated that there exhibited no significant difference in VAS score, sensorimotor block level, duration of labor, mode of delivery, and neonatal safety [38]. Neena et al. studied parturients with spontaneous delivery and normal fetal heart rate [39]. Patients received continuous epidural infusion of 0.0625% bupivacaine + 2 *μ*g/mL fentanyl and 0.1% ropivacaine + 2 *μ*g/mL fentanyl, respectively. 5 ml mixture was added per 5 min. If patients were ineffective within the same initial dose of 15 min, additional drugs were stopped after 90 minutes. The anesthetic effect was evaluated by analgesic effect, motor block, visual analogue scale, maternal hemodynamic parameters, and maternal satisfaction. The results indicated that there exhibited no significant difference. Even so, ropivacaine has become the first choice for epidural labor analgesia because of its sensorimotor separation and low cardiotoxicity.

Sufentanil is a central analgesic, which mainly activates *μ* receptor and has a powerful analgesic effect. Existing studies have found that sufentanil is more lipophilic than fentanyl, and its lipophilicity is about 2 times that of fentanyl and 1000 times that of morphine. Sufentanil can easily pass through nerve cell membrane and blood-brain barrier. Sufentanil has stronger analgesic effect and longer action time, so sufentanil has unique advantages in epidural labor analgesia. During intrathecal administration of sufentanil, it mainly acts on the opioid receptors on the surface of the spinal cord, and its analgesic effect is about 4/5 times higher compared to fentanyl [40]. The high placental transfer rate of sufentanil has raised concerns about the safety of sufentanil, but it has been reported that sufentanil is absorbed into the epidural space very little, so it has little effect on the fetus. Bullingham et al. conducted a randomized, double-blind, placebo-controlled trial involving 400 parturients [41]. All women received epidural anesthesia during the first stage of labor, using 0.08% ropivacaine + 0.4 *μ*g/ml sufentanil, and commonly used patient PCEA control mode. In the second stage of labor, parturients were arbitrarily assigned into two groups and received blind infusion of the same solution or placebo saline, respectively. The main observation index was the duration of the second stage of labor. The results indicated that there exhibited no difference in analgesia score, duration of labor, maternal and neonatal outcome between the placebo group and the epidural group. The results indicated that sufentanil combined with low concentration ropivacaine could not prolong the second stage of labor and could be adopted for epidural labor analgesia. The original research papers published from 1995 to 2014 were searched in order to study which kind of local anesthesia combined with sufentanil was more suitable for epidural labor analgesia. The results of systematic review and meta-analysis indicated that sufentanil combined with bupivacaine, levobupivacaine, and ropivacaine could achieve satisfactory epidural labor analgesia, but the incidence of motor block of bupivacaine-sufentanil was higher. Although the analgesia duration of ropivacaine-sufentanil and levobupivacaine-sufentanil is longer, the rate of instrumental delivery is higher. This study still has some shortcomings. First, the quality of this study is limited due to the small sample size we included in the study. Secondly, this research is a single-center study and our findings are subject to some degree of bias. Therefore, our results may differ from those of large-scale multicenter studies from other academic institutes. This research is still clinically significant and further in-depth investigations will be carried out in the future.

In summary, Sufentanil PCEA combined with ropivacaine for labor analgesia can significantly reduce maternal pain and ensure delivery comfort. Epidural labor analgesia with ropivacaine combined with sufentanil does not reduce fetal umbilical artery blood flow, does not prolong the stage of labor, does not affect the process of delivery and fetal safety, and is safe for parturients and fetuses.

## Figures and Tables

**Table 1:** Comparison of fetal umbilical artery blood flow between the two groups [$\bar{x}$ ± *s*].

Grouping	*N*	PI		RI		S/D	
		Before analgesia	After analgesia	Before analgesia	After analgesia	Before analgesia	After analgesia
Control group	30	0.80 ± 0.22	0.85 ± 0.16	0.55 ± 0.11	0.59 ± 0.12	2.34 ± 0.11	2.59 ± 0.16
Research group	30	0.81 ± 0.21	0.80 ± 0.21	0.56 ± 0.14	0.55 ± 0.21	2.35 ± 0.12	2.54 ± 0.33
*t* value		0.303	1.037	0.307	0.905	0.336	0.746
*P* value		>0.05	>0.05	>0.05	>0.05	>0.05	>0.05

**Table 2:** Comparison of VAS scores between the two groups [$\bar{x}$ ± *s*, points].

Grouping	*N*	Before analgesia	After analgesia 10 min	After analgesia 20 min	After analgesia 30 min
Control group	30	8.59 ± 1.21	6.69 ± 0.31	5.69 ± 1.22	3.55 ± 0.52
Research group	30	8.58 ± 1.22	5.31 ± 1.33	4.31 ± 1.21	2.21 ± 0.31
*t* value		0.031	5.534	4.398	12.123
*P* value		>0.05	<0.05	<0.05	<0.05

**Table 3:** Comparison of the stage of labor and blood loss between the two groups [$\bar{x}$ ± *s*].

Grouping	*N*	The first stage of labor (min)	The second stage of labor (min)	The third stage of labor (min)	Bleeding volume (ml)
Control group	30	445.81 ± 24.22	50.19 ± 3.66	24.91 ± 2.33	280.95 ± 25.44
Research group	30	430.19 ± 12.45	45.91 ± 4.32	20.91 ± 2.34	265.95 ± 20.52
*t* value		2.698	4.140	6.634	2.513
*P* value		<0.05	<0.05	<0.05	<0.05

**Table 4:** Comparison of Apgar score between two groups of newborns [$\bar{x}$ ± *s*, points].

Grouping	*N*	Birth 1 min Apgar scoring	Birth 5 min Apgar scoring	Birth 10 min Apgar scoring
Control group	30	9.01 ± 0.01	9.45 ± 0.12	9.82 ± 0.01
Research group	30	9.45 ± 0.44	10.00 ± 0.00	10.00 ± 0.00
*t* value		5.475	25.103	98.590
*P* value		<0.05	<0.05	<0.05

## Data Availability

The data sets used and analyzed during the current study are available from the corresponding author upon reasonable request.
